# Supplementary material for: Crossroads between Autoimmunity and COVID-19 in Lung Transplant Recipients
Source: Viruses. 2023 Oct 3;15(10):2045. doi: 10.3390/v15102045 (PMC10612071; doi:10.3390/v15102045)
Supplement: Supplementary file 1 [file viruses-15-02045-s001.zip › viruses-2596551-supplementary.pptx]

## Slide 1
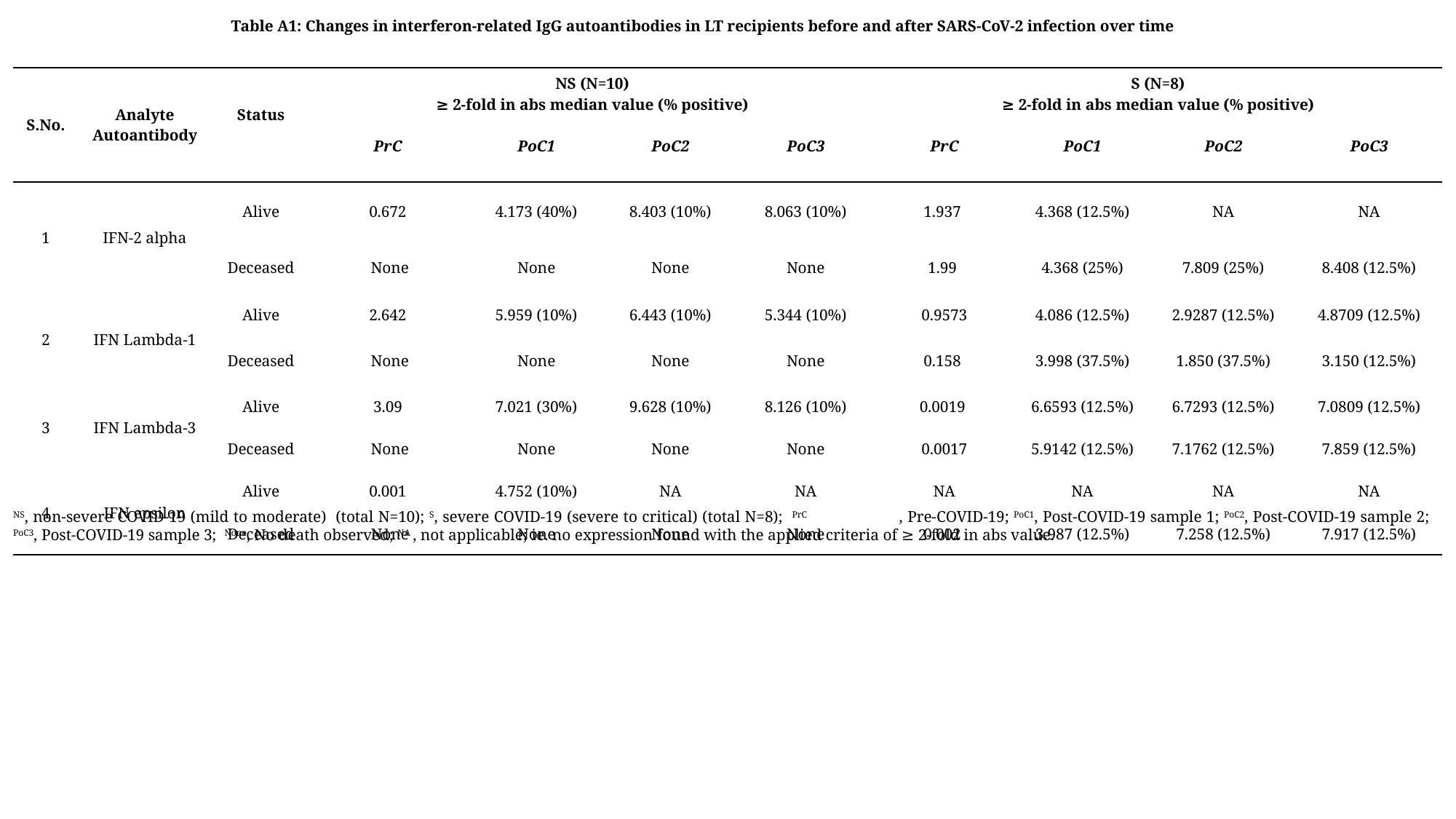

Table A1: Changes in interferon-related IgG autoantibodies in LT recipients before and after SARS-CoV-2 infection over time
| S.No. | Analyte Autoantibody | Status | NS (N=10) ≥ 2-fold in abs median value (% positive) | | | | S (N=8) ≥ 2-fold in abs median value (% positive) | | | |
| --- | --- | --- | --- | --- | --- | --- | --- | --- | --- | --- |
| | | | PrC | PoC1 | PoC2 | PoC3 | PrC | PoC1 | PoC2 | PoC3 |
| 1 | IFN-2 alpha | Alive | 0.672 | 4.173 (40%) | 8.403 (10%) | 8.063 (10%) | 1.937 | 4.368 (12.5%) | NA | NA |
| | | Deceased | None | None | None | None | 1.99 | 4.368 (25%) | 7.809 (25%) | 8.408 (12.5%) |
| 2 | IFN Lambda-1 | Alive | 2.642 | 5.959 (10%) | 6.443 (10%) | 5.344 (10%) | 0.9573 | 4.086 (12.5%) | 2.9287 (12.5%) | 4.8709 (12.5%) |
| | | Deceased | None | None | None | None | 0.158 | 3.998 (37.5%) | 1.850 (37.5%) | 3.150 (12.5%) |
| 3 | IFN Lambda-3 | Alive | 3.09 | 7.021 (30%) | 9.628 (10%) | 8.126 (10%) | 0.0019 | 6.6593 (12.5%) | 6.7293 (12.5%) | 7.0809 (12.5%) |
| | | Deceased | None | None | None | None | 0.0017 | 5.9142 (12.5%) | 7.1762 (12.5%) | 7.859 (12.5%) |
| 4 | IFN epsilon | Alive | 0.001 | 4.752 (10%) | NA | NA | NA | NA | NA | NA |
| | | Deceased | None | None | None | None | 0.002 | 3.987 (12.5%) | 7.258 (12.5%) | 7.917 (12.5%) |
NS, non-severe COVID-19 (mild to moderate) (total N=10); S, severe COVID-19 (severe to critical) (total N=8); PrC	, Pre-COVID-19; PoC1, Post-COVID-19 sample 1; PoC2, Post-COVID-19 sample 2; PoC3, Post-COVID-19 sample 3; None, No death observed; NA , not applicable; ie. no expression found with the applied criteria of ≥ 2-fold in abs value.

## Slide 2
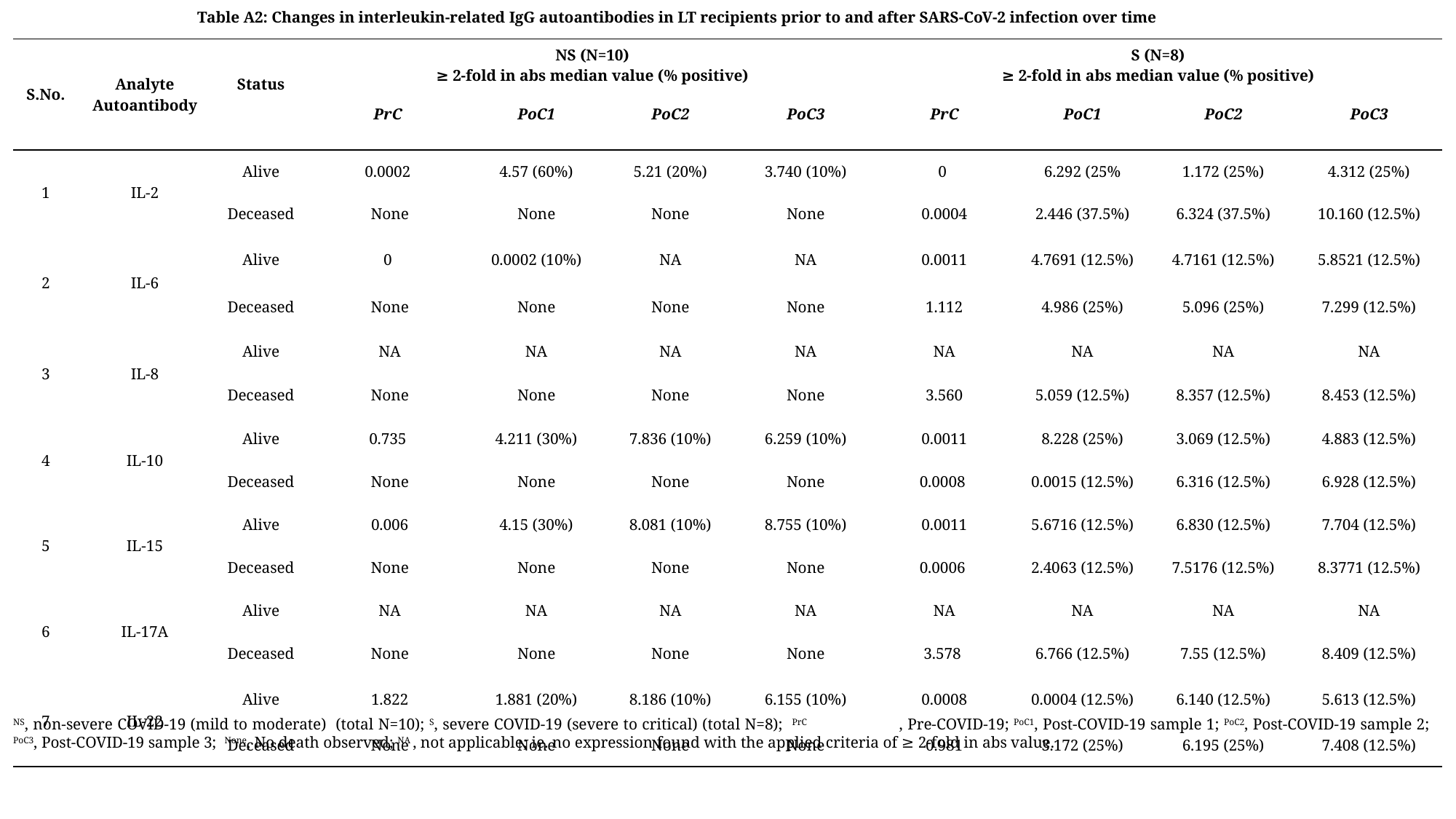

Table A2: Changes in interleukin-related IgG autoantibodies in LT recipients prior to and after SARS-CoV-2 infection over time
| S.No. | Analyte Autoantibody | Status | NS (N=10) ≥ 2-fold in abs median value (% positive) | | | | S (N=8) ≥ 2-fold in abs median value (% positive) | | | |
| --- | --- | --- | --- | --- | --- | --- | --- | --- | --- | --- |
| | | | PrC | PoC1 | PoC2 | PoC3 | PrC | PoC1 | PoC2 | PoC3 |
| 1 | IL-2 | Alive | 0.0002 | 4.57 (60%) | 5.21 (20%) | 3.740 (10%) | 0 | 6.292 (25% | 1.172 (25%) | 4.312 (25%) |
| | | Deceased | None | None | None | None | 0.0004 | 2.446 (37.5%) | 6.324 (37.5%) | 10.160 (12.5%) |
| 2 | IL-6 | Alive | 0 | 0.0002 (10%) | NA | NA | 0.0011 | 4.7691 (12.5%) | 4.7161 (12.5%) | 5.8521 (12.5%) |
| | | Deceased | None | None | None | None | 1.112 | 4.986 (25%) | 5.096 (25%) | 7.299 (12.5%) |
| 3 | IL-8 | Alive | NA | NA | NA | NA | NA | NA | NA | NA |
| | | Deceased | None | None | None | None | 3.560 | 5.059 (12.5%) | 8.357 (12.5%) | 8.453 (12.5%) |
| 4 | IL-10 | Alive | 0.735 | 4.211 (30%) | 7.836 (10%) | 6.259 (10%) | 0.0011 | 8.228 (25%) | 3.069 (12.5%) | 4.883 (12.5%) |
| | | Deceased | None | None | None | None | 0.0008 | 0.0015 (12.5%) | 6.316 (12.5%) | 6.928 (12.5%) |
| 5 | IL-15 | Alive | 0.006 | 4.15 (30%) | 8.081 (10%) | 8.755 (10%) | 0.0011 | 5.6716 (12.5%) | 6.830 (12.5%) | 7.704 (12.5%) |
| | | Deceased | None | None | None | None | 0.0006 | 2.4063 (12.5%) | 7.5176 (12.5%) | 8.3771 (12.5%) |
| 6 | IL-17A | Alive | NA | NA | NA | NA | NA | NA | NA | NA |
| | | Deceased | None | None | None | None | 3.578 | 6.766 (12.5%) | 7.55 (12.5%) | 8.409 (12.5%) |
| 7 | IL-22 | Alive | 1.822 | 1.881 (20%) | 8.186 (10%) | 6.155 (10%) | 0.0008 | 0.0004 (12.5%) | 6.140 (12.5%) | 5.613 (12.5%) |
| | | Deceased | None | None | None | None | 0.981 | 3.172 (25%) | 6.195 (25%) | 7.408 (12.5%) |
NS, non-severe COVID-19 (mild to moderate) (total N=10); S, severe COVID-19 (severe to critical) (total N=8); PrC	, Pre-COVID-19; PoC1, Post-COVID-19 sample 1; PoC2, Post-COVID-19 sample 2; PoC3, Post-COVID-19 sample 3; None, No death observed; NA , not applicable; ie. no expression found with the applied criteria of ≥ 2-fold in abs value.

## Slide 3
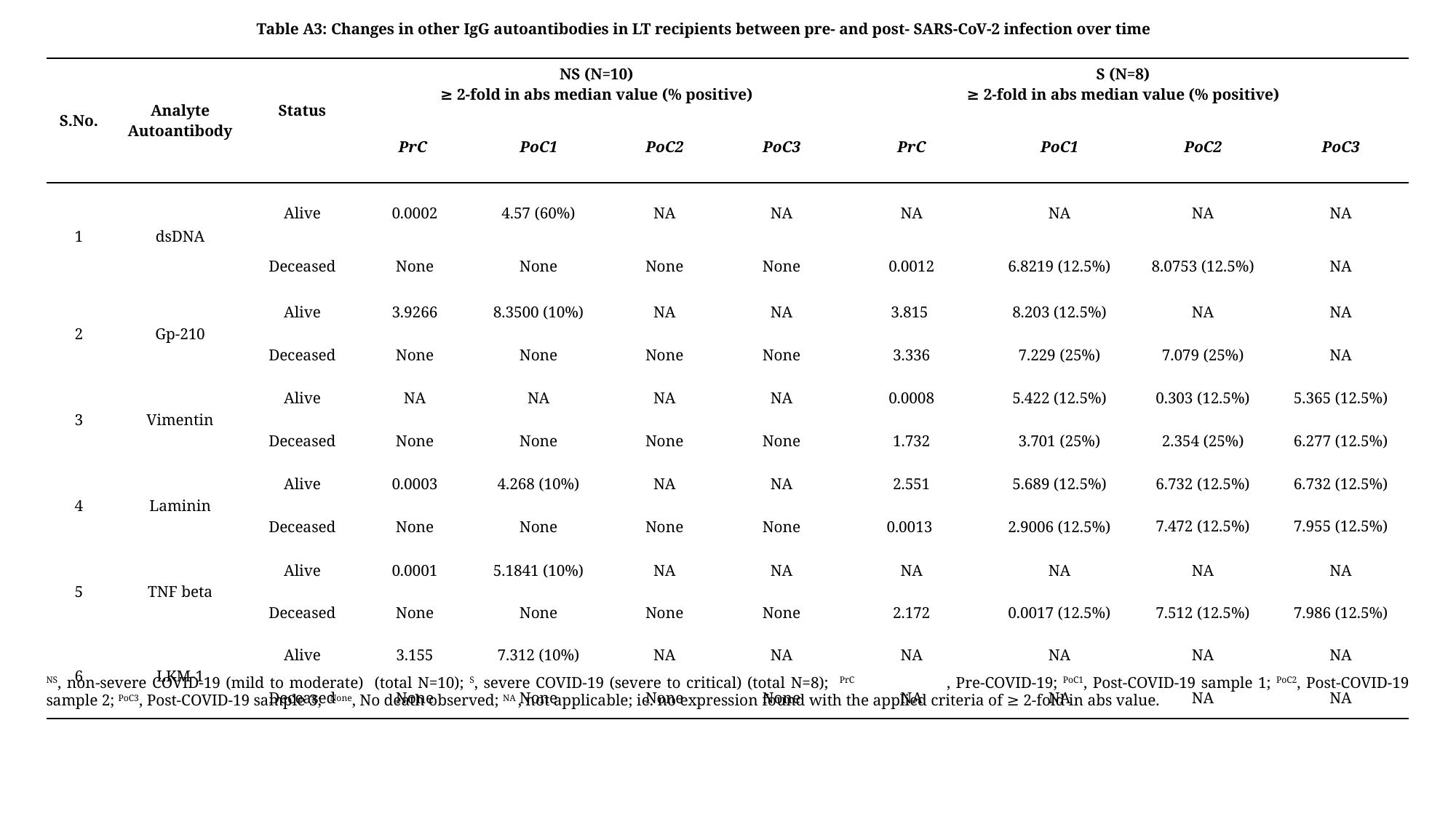

Table A3: Changes in other IgG autoantibodies in LT recipients between pre- and post- SARS-CoV-2 infection over time
| S.No. | Analyte Autoantibody | Status | NS (N=10) ≥ 2-fold in abs median value (% positive) | | | | S (N=8) ≥ 2-fold in abs median value (% positive) | | | |
| --- | --- | --- | --- | --- | --- | --- | --- | --- | --- | --- |
| | | | PrC | PoC1 | PoC2 | PoC3 | PrC | PoC1 | PoC2 | PoC3 |
| 1 | dsDNA | Alive | 0.0002 | 4.57 (60%) | NA | NA | NA | NA | NA | NA |
| | | Deceased | None | None | None | None | 0.0012 | 6.8219 (12.5%) | 8.0753 (12.5%) | NA |
| 2 | Gp-210 | Alive | 3.9266 | 8.3500 (10%) | NA | NA | 3.815 | 8.203 (12.5%) | NA | NA |
| | | Deceased | None | None | None | None | 3.336 | 7.229 (25%) | 7.079 (25%) | NA |
| 3 | Vimentin | Alive | NA | NA | NA | NA | 0.0008 | 5.422 (12.5%) | 0.303 (12.5%) | 5.365 (12.5%) |
| | | Deceased | None | None | None | None | 1.732 | 3.701 (25%) | 2.354 (25%) | 6.277 (12.5%) |
| 4 | Laminin | Alive | 0.0003 | 4.268 (10%) | NA | NA | 2.551 | 5.689 (12.5%) | 6.732 (12.5%) | 6.732 (12.5%) |
| | | Deceased | None | None | None | None | 0.0013 | 2.9006 (12.5%) | 7.472 (12.5%) | 7.955 (12.5%) |
| 5 | TNF beta | Alive | 0.0001 | 5.1841 (10%) | NA | NA | NA | NA | NA | NA |
| | | Deceased | None | None | None | None | 2.172 | 0.0017 (12.5%) | 7.512 (12.5%) | 7.986 (12.5%) |
| 6 | LKM-1 | Alive | 3.155 | 7.312 (10%) | NA | NA | NA | NA | NA | NA |
| | | Deceased | None | None | None | None | NA | NA | NA | NA |
NS, non-severe COVID-19 (mild to moderate) (total N=10); S, severe COVID-19 (severe to critical) (total N=8); PrC	, Pre-COVID-19; PoC1, Post-COVID-19 sample 1; PoC2, Post-COVID-19 sample 2; PoC3, Post-COVID-19 sample 3; None, No death observed; NA , not applicable; ie. no expression found with the applied criteria of ≥ 2-fold in abs value.

## Slide 4
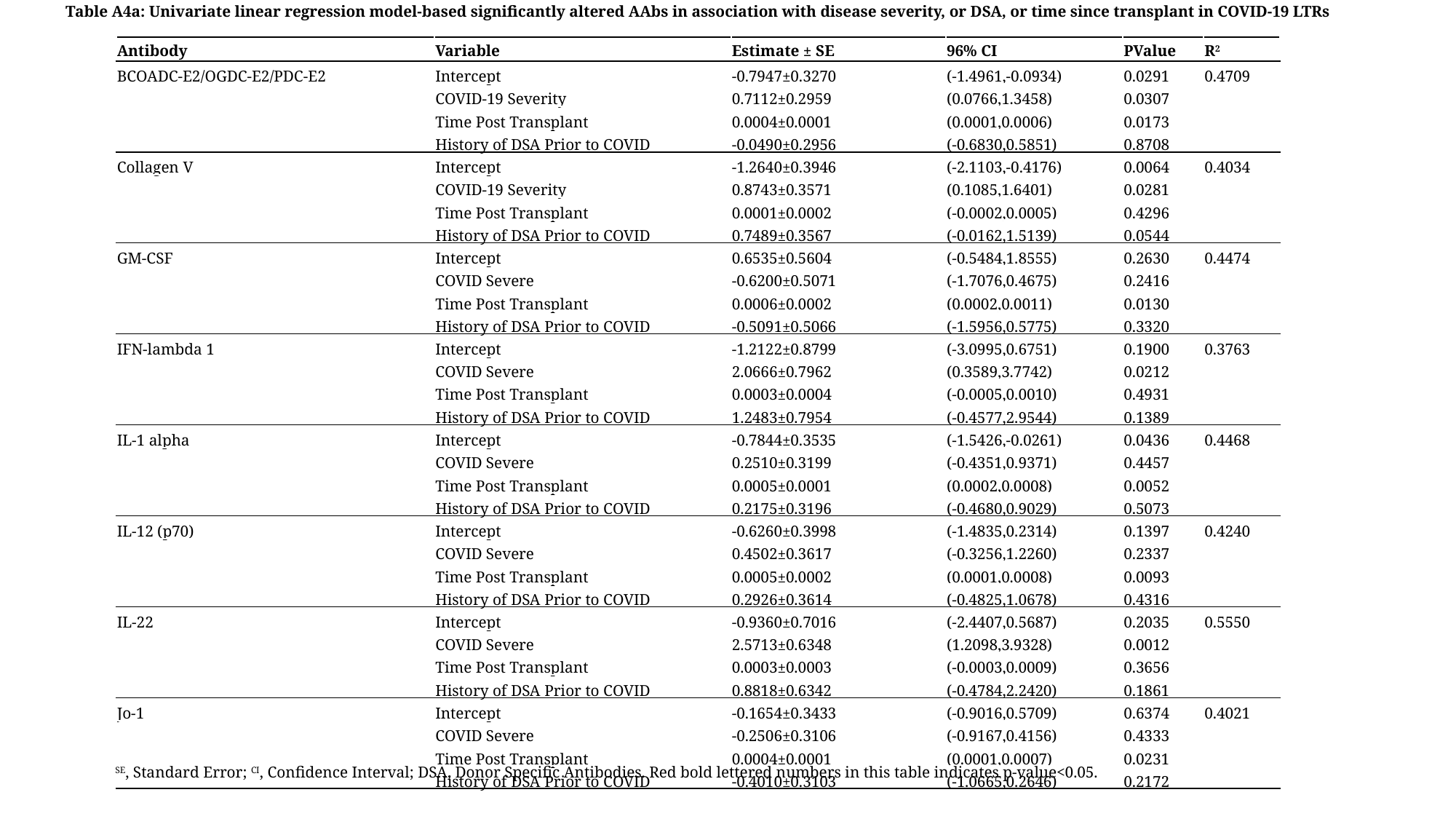

Table A4a: Univariate linear regression model-based significantly altered AAbs in association with disease severity, or DSA, or time since transplant in COVID-19 LTRs
| Antibody | Variable | Estimate ± SE | 96% CI | PValue | R2 |
| --- | --- | --- | --- | --- | --- |
| BCOADC-E2/OGDC-E2/PDC-E2 | Intercept | -0.7947±0.3270 | (-1.4961,-0.0934) | 0.0291 | 0.4709 |
| | COVID-19 Severity | 0.7112±0.2959 | (0.0766,1.3458) | 0.0307 | |
| | Time Post Transplant | 0.0004±0.0001 | (0.0001,0.0006) | 0.0173 | |
| | History of DSA Prior to COVID | -0.0490±0.2956 | (-0.6830,0.5851) | 0.8708 | |
| Collagen V | Intercept | -1.2640±0.3946 | (-2.1103,-0.4176) | 0.0064 | 0.4034 |
| | COVID-19 Severity | 0.8743±0.3571 | (0.1085,1.6401) | 0.0281 | |
| | Time Post Transplant | 0.0001±0.0002 | (-0.0002,0.0005) | 0.4296 | |
| | History of DSA Prior to COVID | 0.7489±0.3567 | (-0.0162,1.5139) | 0.0544 | |
| GM-CSF | Intercept | 0.6535±0.5604 | (-0.5484,1.8555) | 0.2630 | 0.4474 |
| | COVID Severe | -0.6200±0.5071 | (-1.7076,0.4675) | 0.2416 | |
| | Time Post Transplant | 0.0006±0.0002 | (0.0002,0.0011) | 0.0130 | |
| | History of DSA Prior to COVID | -0.5091±0.5066 | (-1.5956,0.5775) | 0.3320 | |
| IFN-lambda 1 | Intercept | -1.2122±0.8799 | (-3.0995,0.6751) | 0.1900 | 0.3763 |
| | COVID Severe | 2.0666±0.7962 | (0.3589,3.7742) | 0.0212 | |
| | Time Post Transplant | 0.0003±0.0004 | (-0.0005,0.0010) | 0.4931 | |
| | History of DSA Prior to COVID | 1.2483±0.7954 | (-0.4577,2.9544) | 0.1389 | |
| IL-1 alpha | Intercept | -0.7844±0.3535 | (-1.5426,-0.0261) | 0.0436 | 0.4468 |
| | COVID Severe | 0.2510±0.3199 | (-0.4351,0.9371) | 0.4457 | |
| | Time Post Transplant | 0.0005±0.0001 | (0.0002,0.0008) | 0.0052 | |
| | History of DSA Prior to COVID | 0.2175±0.3196 | (-0.4680,0.9029) | 0.5073 | |
| IL-12 (p70) | Intercept | -0.6260±0.3998 | (-1.4835,0.2314) | 0.1397 | 0.4240 |
| | COVID Severe | 0.4502±0.3617 | (-0.3256,1.2260) | 0.2337 | |
| | Time Post Transplant | 0.0005±0.0002 | (0.0001,0.0008) | 0.0093 | |
| | History of DSA Prior to COVID | 0.2926±0.3614 | (-0.4825,1.0678) | 0.4316 | |
| IL-22 | Intercept | -0.9360±0.7016 | (-2.4407,0.5687) | 0.2035 | 0.5550 |
| | COVID Severe | 2.5713±0.6348 | (1.2098,3.9328) | 0.0012 | |
| | Time Post Transplant | 0.0003±0.0003 | (-0.0003,0.0009) | 0.3656 | |
| | History of DSA Prior to COVID | 0.8818±0.6342 | (-0.4784,2.2420) | 0.1861 | |
| Jo-1 | Intercept | -0.1654±0.3433 | (-0.9016,0.5709) | 0.6374 | 0.4021 |
| | COVID Severe | -0.2506±0.3106 | (-0.9167,0.4156) | 0.4333 | |
| | Time Post Transplant | 0.0004±0.0001 | (0.0001,0.0007) | 0.0231 | |
| | History of DSA Prior to COVID | -0.4010±0.3103 | (-1.0665,0.2646) | 0.2172 | |
SE, Standard Error; CI, Confidence Interval; DSA, Donor Specific Antibodies. Red bold lettered numbers in this table indicates p-value<0.05.

## Slide 5
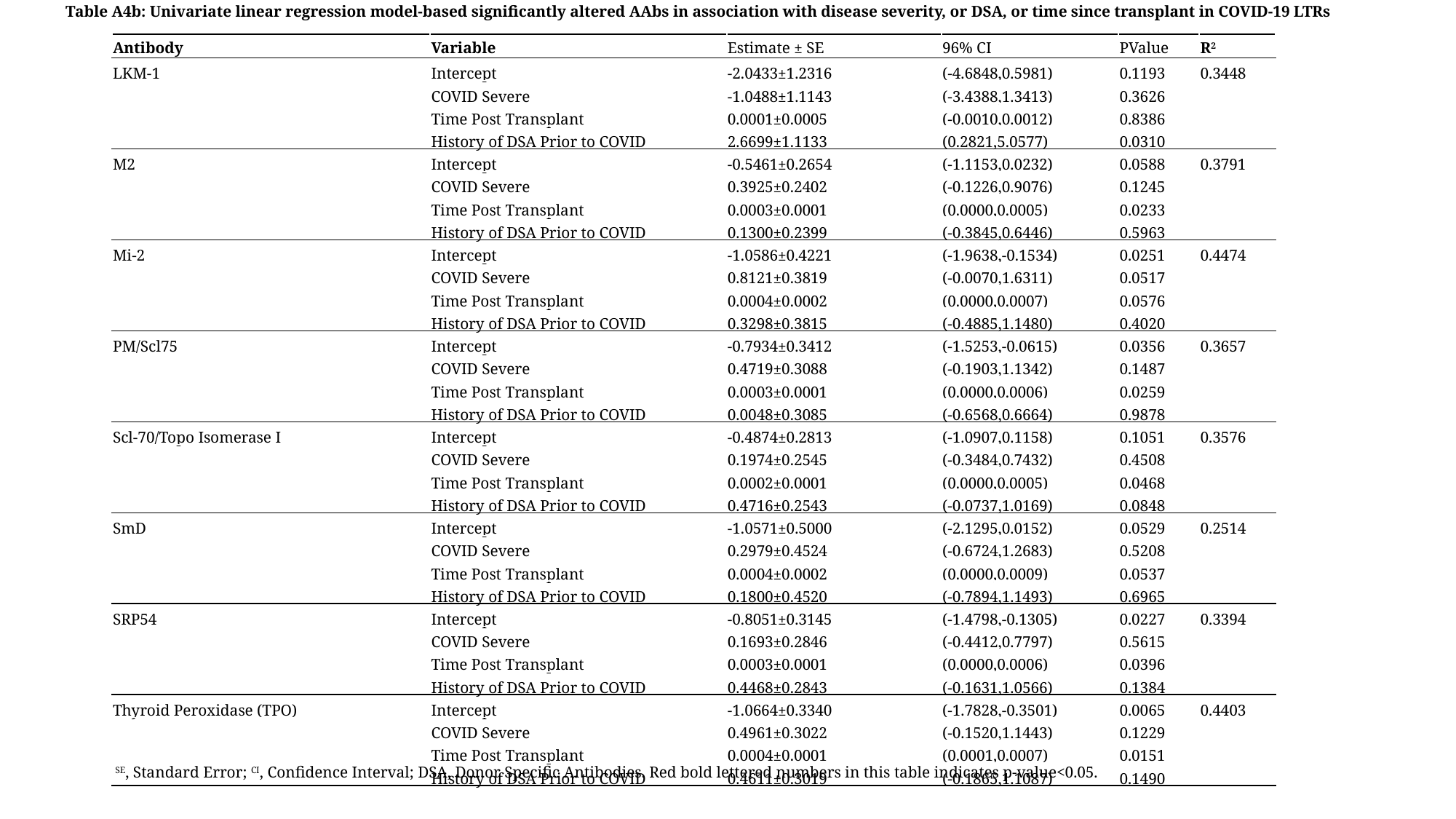

Table A4b: Univariate linear regression model-based significantly altered AAbs in association with disease severity, or DSA, or time since transplant in COVID-19 LTRs
| Antibody | Variable | Estimate ± SE | 96% CI | PValue | R2 |
| --- | --- | --- | --- | --- | --- |
| LKM-1 | Intercept | -2.0433±1.2316 | (-4.6848,0.5981) | 0.1193 | 0.3448 |
| | COVID Severe | -1.0488±1.1143 | (-3.4388,1.3413) | 0.3626 | |
| | Time Post Transplant | 0.0001±0.0005 | (-0.0010,0.0012) | 0.8386 | |
| | History of DSA Prior to COVID | 2.6699±1.1133 | (0.2821,5.0577) | 0.0310 | |
| M2 | Intercept | -0.5461±0.2654 | (-1.1153,0.0232) | 0.0588 | 0.3791 |
| | COVID Severe | 0.3925±0.2402 | (-0.1226,0.9076) | 0.1245 | |
| | Time Post Transplant | 0.0003±0.0001 | (0.0000,0.0005) | 0.0233 | |
| | History of DSA Prior to COVID | 0.1300±0.2399 | (-0.3845,0.6446) | 0.5963 | |
| Mi-2 | Intercept | -1.0586±0.4221 | (-1.9638,-0.1534) | 0.0251 | 0.4474 |
| | COVID Severe | 0.8121±0.3819 | (-0.0070,1.6311) | 0.0517 | |
| | Time Post Transplant | 0.0004±0.0002 | (0.0000,0.0007) | 0.0576 | |
| | History of DSA Prior to COVID | 0.3298±0.3815 | (-0.4885,1.1480) | 0.4020 | |
| PM/Scl75 | Intercept | -0.7934±0.3412 | (-1.5253,-0.0615) | 0.0356 | 0.3657 |
| | COVID Severe | 0.4719±0.3088 | (-0.1903,1.1342) | 0.1487 | |
| | Time Post Transplant | 0.0003±0.0001 | (0.0000,0.0006) | 0.0259 | |
| | History of DSA Prior to COVID | 0.0048±0.3085 | (-0.6568,0.6664) | 0.9878 | |
| Scl-70/Topo Isomerase I | Intercept | -0.4874±0.2813 | (-1.0907,0.1158) | 0.1051 | 0.3576 |
| | COVID Severe | 0.1974±0.2545 | (-0.3484,0.7432) | 0.4508 | |
| | Time Post Transplant | 0.0002±0.0001 | (0.0000,0.0005) | 0.0468 | |
| | History of DSA Prior to COVID | 0.4716±0.2543 | (-0.0737,1.0169) | 0.0848 | |
| SmD | Intercept | -1.0571±0.5000 | (-2.1295,0.0152) | 0.0529 | 0.2514 |
| | COVID Severe | 0.2979±0.4524 | (-0.6724,1.2683) | 0.5208 | |
| | Time Post Transplant | 0.0004±0.0002 | (0.0000,0.0009) | 0.0537 | |
| | History of DSA Prior to COVID | 0.1800±0.4520 | (-0.7894,1.1493) | 0.6965 | |
| SRP54 | Intercept | -0.8051±0.3145 | (-1.4798,-0.1305) | 0.0227 | 0.3394 |
| | COVID Severe | 0.1693±0.2846 | (-0.4412,0.7797) | 0.5615 | |
| | Time Post Transplant | 0.0003±0.0001 | (0.0000,0.0006) | 0.0396 | |
| | History of DSA Prior to COVID | 0.4468±0.2843 | (-0.1631,1.0566) | 0.1384 | |
| Thyroid Peroxidase (TPO) | Intercept | -1.0664±0.3340 | (-1.7828,-0.3501) | 0.0065 | 0.4403 |
| | COVID Severe | 0.4961±0.3022 | (-0.1520,1.1443) | 0.1229 | |
| | Time Post Transplant | 0.0004±0.0001 | (0.0001,0.0007) | 0.0151 | |
| | History of DSA Prior to COVID | 0.4611±0.3019 | (-0.1865,1.1087) | 0.1490 | |
SE, Standard Error; CI, Confidence Interval; DSA, Donor Specific Antibodies. Red bold lettered numbers in this table indicates p-value<0.05.
